# Yield-quality relationships under deficit irrigation in greenhouse tomato: modulation by seaweed biostimulants

**DOI:** 10.3389/fpls.2026.1859338

**Published:** 2026-05-26

**Authors:** Ivanka Tringovska, Gergana Marovska, Georgi Velichkov, Veneta Stoeva, Viktoria Atanasova, Delcho Dobrev, Daniela Ganeva, Stanislava Grozeva

**Affiliations:** 1 Maritsa Vegetable Crops Research Institute, Agricultural Academy, Plovdiv, Bulgaria; 2 Agriflor Ltd., Breznik, Bulgaria

**Keywords:** deficit irrigation, fruit composition, greenhouse tomato, seaweed biostimulants, soil moisture, temporal yield distribution, yield components, yield-quality relationship

## Abstract

**Introduction:**

Water availability is a crucial factor for crop performance in greenhouse tomato production, affecting both yield development and fruit composition traits. This study examined how deficit irrigation alters the relationship between yield and quality in tomatoes and whether foliar application of seaweed-derived biostimulants impacts these responses.

**Methods:**

A greenhouse experiment was carried out under two different irrigation levels, characterized by soil moisture contents of approximately 23–27% under optimal watering and 12–15% under reduced watering, using treatments based on extracts of Laminaria digitata and Ascophyllum nodosum, applied separately and together. Yield components, temporal yield dynamics, and concentration-related compositional traits were evaluated, and integrative PCA and concentration-index analyses were performed.

**Results:**

Reduced irrigation altered the yield structure by increasing fruit number and decreasing average fruit weight, while total yield showed limited variation among treatments. At the same time, concentration-related traits, including dry matter, soluble solids, vitamin C, and titratable acidity, were consistently higher under reduced irrigation. Biostimulant effects were dependent on the irrigation regime. Under optimal irrigation, treatment effects were limited, whereas under reduced irrigation, some treatments were associated with higher yields, primarily due to differences in fruit number during the main production peak. However, these yield responses were not consistently aligned with changes in compositional traits. Temporal yield analysis further showed that treatment effects under reduced irrigation were expressed mainly through modifications in the timing and intensity of peak production rather than through uniform effects across the entire production cycle. Integrative PCA and concentration-index analysis indicated that compositional enhancement and yield performance were only partly associated under reduced irrigation, suggesting a partial decoupling between productivity and fruit quality responses.

**Discussion:**

Overall, the results show that the irrigation regime is the main factor influencing yield–quality relationships in greenhouse tomato, while biostimulant treatments add extra, context-dependent variability. These findings emphasize the importance of evaluating both yield components and compositional traits together when analyzing crop responses under limited water availability.

## 1 Introduction

Water availability plays a key role in crop performance in greenhouse vegetable production, affecting both yield development and fruit quality traits. In tomato, deficit irrigation is commonly used as a management tool to improve resource efficiency and boost certain quality parameters. However, lower water supply usually alters source–sink relationships, resulting in changes in yield components such as fruit number and average fruit weight, along with variations in fruit biochemical composition (Patanè and Cosentino, 2010; Ripoll et al., 2014, 2016; Lovelli et al., 2017). Although increases in dry matter, soluble solids, organic acids, and antioxidants are often observed under water stress, these changes may be due to a mix of concentration effects from reduced fruit water content and stress-driven metabolic adjustments (Guichard et al., 2001; Ripoll et al., 2014).

The relationship between yield and fruit composition under deficit irrigation is therefore complex. Moderate water limitation can enhance compositional traits with minimal impact on yield, while more severe constraints might cause trade-offs between productivity and quality, depending on stress level, timing, and genotype (Ripoll et al., 2014, 2016). Understanding how yield components and compositional traits vary together under different water regimes is essential for optimizing irrigation strategies in greenhouse systems.

In this context, plant biostimulants are seen as tools to modify how plants respond to less-than-ideal environmental conditions. Seaweed-based products, especially those derived from *Ascophyllum nodosum* and *Laminaria digitata*, have been reported to affect plant growth, nutrient absorption, and responses to stress (Calvo et al., 2014; Rouphael and Colla, 2020; Shukla et al., 2019; Ali et al., 2021). However, their effectiveness heavily depends on environmental factors, and understanding their role during deficit irrigation remains challenging. Particularly, it is often unclear whether changes in fruit composition seen with combined irrigation and biostimulant treatments indicate improved physiological performance or are mainly caused by water-deficit concentration effects.

Therefore, a key question is whether biostimulant application alters the relationship between yield formation and fruit compositional traits under different irrigation regimes. Instead of focusing only on absolute changes in yield or individual quality parameters, an integrated assessment of yield components and compositional responses may give a more functionally relevant understanding of treatment effects.

The aim of this study was to evaluate how deficit irrigation affects yield–quality relationships in greenhouse-grown tomato and to determine whether foliar application of seaweed-derived biostimulants alters these responses. We hypothesized that (i) deficit irrigation would be the main factor influencing fruit composition, and (ii) biostimulant effects would be dependent on context, impacting yield development and its link with compositional traits without necessarily resulting in uniform improvements across all parameters.

## 2 Materials and methods

### 2.1 Experimental site and plant material

The experiment was conducted during the spring-summer season of 2025 in an unheated Venlo type steel-glass greenhouse at the Maritsa Vegetable Crops Research Institute, Plovdiv, Bulgaria (42.177867, 24.762276).

Tomato (*Solanum lycopersicum* L.) cv. ‘Mami Pink’ (Enza Zaden) was used. Seeds were sown on 10 January, seedlings were pricked-out into trays on 18 February, and transplanted in the greenhouse on 24 March at a density of 3.0 plants m^−2^. Harvesting continued until 30 July.

Plants were grown directly in greenhouse soil, following standard commercial practices for indeterminate tomato production. All treatments received the same agronomic management, including fertigation, pruning, plant protection, and canopy management. The growing tip was removed above the fifth inflorescence.

### 2.2 Experimental design

The experiment was arranged as a factorial experiment with two factors (irrigation regime and biostimulant treatment):

- Factor A: Irrigation regime: Optimal irrigation and Reduced irrigation (water deficit)
- Factor B: Biostimulant treatment
- Control (no foliar treatment)
- *Laminaria digitata* – 0.7%
- *Laminaria digitata* – 1.0%
- *Ascophyllum nodosum* – 0.7%
- *Ascophyllum nodosum* – 1.0%
- *L. digitata + A. nodosum* – 0.7%
- *L. digitata + A. nodosum* – 1.0%

The treatments were arranged using a randomized block method with three replications.

### 2.3 Irrigation regimes and environmental conditions

Two irrigation regimes were established to create different soil water conditions: optimal irrigation and reduced irrigation. Due to technical limits of the greenhouse facility, the two regimes were carried out in separate but structurally similar compartments (GH9 and GH8), which followed the same agronomic practices, plant density, fertilization, and crop management protocols. Nevertheless, the compartments differed in microclimatic conditions, particularly in air temperature and relative humidity, as a consequence of the different irrigation regimes and associated soil-atmosphere interactions. Because each irrigation regime was imposed in a single greenhouse compartment, irrigation was applied at the compartment level and therefore lacked independent compartment-level replication. The irrigation treatments were initiated in mid-May, after plant establishment and at flowering and first fruit set, and continued throughout the main fruit development and harvesting period until July.

Soil moisture, soil temperature, electrical conductivity (EC), air temperature, and relative humidity were continuously monitored throughout the experimental period (May–July) using the installed sensor network. Soil parameters were monitored using EnviroPro EP100G-04 sensors (Entelechy Pty Ltd.), providing measurements at 10, 20, 30, and 40 cm soil depths, while air temperature and relative humidity were monitored using AM2302 sensors (AOSONG) installed at 50, 100, and 150 cm above the soil surface. Soil moisture values were expressed as volumetric water content (VWC, % v/v). Sensors were factory-calibrated according to manufacturer specifications. Under optimal irrigation, soil moisture was maintained within a relatively stable range of approximately 23–27%, whereas under reduced irrigation it consistently ranged between 12–15%, indicating a sustained reduction in water availability in the root zone. Temporal dynamics of soil moisture, soil temperature, EC, air temperature, and relative humidity during the experimental period are presented in **Supplementary Figure 1**.

Besides soil moisture differences, the reduced irrigation compartment showed slightly higher air temperatures and lower relative humidity compared to the optimal irrigation compartment, reflecting the coupled soil-atmosphere responses to decreased water input. These environmental changes are typical of irrigation management in greenhouse conditions and are frequently seen in commercial production systems.

Therefore, the two irrigation regimes represented distinct and agriculturally relevant irrigation environments, incorporating both soil and atmospheric responses to water availability. This approach offers a practical framework for assessing plant performance under different water supply conditions, as it captures the combined effects of irrigation on the crop microclimate rather than focusing solely on soil moisture.

### 2.4 Fertilization and crop management

Before transplanting, basal fertilization was applied to supply the following active ingredients: 5.4 kg N da^−1^; 24.9 kg P_2_O_5_ da^−1^ and 16.9–17.5 kg K_2_O da^−1^. Nitrogen was provided through organic and mineral sources, phosphorus predominantly through triple superphosphate, and potassium through potassium sulfate and organic fertilizers. Basal fertilization was incorporated into the soil prior to planting.

During the growing period, vegetative fertilization was applied via fertigation using water-soluble fertilizers. Total seasonal nutrient supply amounted to 17 kg da^−1^ N, 13 kg da^−1^ P_2_O_5_, and 43 kg da^−1^ K_2_O. Fertigation was adjusted according to plant developmental stage and irrigation regime.

All treatments received identical agronomic practices, including irrigation scheduling (according to treatment), fertigation management, plant protection measures, pruning, and canopy management. The growing tip was removed above the fifth inflorescence.

### 2.5 Biostimulant products and application

Seaweed extracts derived from the brown macroalgae *Ascophyllum nodosum* (*Fucaceae*) and *Laminaria digitata* (*Laminariaceae*) were used. The products were obtained through cold extraction without organic solvents or alkaline hydrolysis and formulated with non-toxic co-formulants. According to the manufacturer, the products contained naturally occurring polysaccharides, organic acids, amino acids, mineral nutrients, and low concentrations of phytohormone-like compounds typically associated with brown seaweed extracts. All applications were performed using products from the same commercial batches to minimize batch-to-batch variability.

Three foliar applications were performed during flowering (12 May-5 June) at 10–12-day intervals. Solutions were prepared at concentrations of 0.7% and 1.0% (v/v), according to treatment. Foliar applications were carried out in the early morning under greenhouse conditions to minimize rapid evaporation and improve leaf surface retention. Approximately 1.2 L of spray solution was applied per treatment replicate, corresponding to approximately 50 mL per plant, although the effective spray volume varied depending on canopy development stage to ensure uniform leaf wetting without excessive runoff.

### 2.6 Research parameters

Yield components – number of fruits per plant and average fruit weight were evaluated at each harvest.

Total yield – was calculated based on cumulative fruit weight per experimental unit and expressed per unit area.

Fruit quality analyses were performed using fruits collected at the third harvest, corresponding to the period of stable commercial production under both irrigation environments. This harvest was selected to minimize the greater variability commonly associated with the earliest and latest harvests. Fruits were sampled at the red-ripe commercial stage on July, 4th. The following were assessed:

- Biochemical parameters: Dry matter (%) – gravimetric method; Soluble solids (°Brix) – refractometric method; Vitamin C (mg 100 g^−1^ FW) – titrimetric method by Tillmans reaction; pH – potentiometric measurement; Titratable organic acids (% citric acid) – titrimetric method
- Ionic composition: NO_3_^−^, Na^+^, Ca²^+^, K^+^ and Electrical conductivity – measured using HORIBA LAQUA ion-selective electrodes. The selected ionic parameters served as complementary indicators of fruit compositional quality and ion balance across different irrigation conditions.;
- Physical parameters: Fruit firmness (°Shore) – digital durometer; Fruit color (L*, a*, b*, Chroma, Hue) – chromameter

### 2.7 Statistical analyses

Data were analyzed using general linear models including irrigation environment, biostimulant treatment, and their interaction as fixed factors. Temporal yield dynamics across harvests were analyzed using general linear models including irrigation environment, biostimulant treatment, and harvest timing as fixed factors. Interaction terms were not retained in the final harvest-dynamics models because of structural imbalance among some harvest combinations. Mean comparisons were performed using Tukey’s HSD test at p ≤ 0.05. Statistical significance levels were considered at p < 0.05, p < 0.01, and p < 0.001. Assumptions of normality and homogeneity of variance were evaluated prior to analysis. Because irrigation regimes were imposed at the greenhouse-compartment level, independent compartment-level replication of the irrigation factor was not available. Therefore, irrigation-related effects were interpreted cautiously as differences between greenhouse irrigation environments rather than as fully replicated irrigation treatment effects. Hierarchical clustering analysis was applied to standardized fruit compositional and mineral parameters to identify treatment-dependent patterns within each irrigation regime. For each regime, treatment means were normalized to z-scores. Pairwise distances were calculated using Euclidean distance, and clustering was performed using complete linkage. Heatmaps were generated in R using the pheatmap package, with rows and columns ordered by hierarchical clustering.

Principal component analysis (PCA) was performed on standardized treatment means (irrigation × treatment combinations) to integrate yield, fruit number, compositional and mineral traits. Variables were centered and scaled prior to analysis, and components were extracted from the correlation matrix using singular value decomposition (prcomp, R). Explained variance was calculated from eigenvalues, and variable contributions were derived from squared loadings.

All statistical analyses were conducted using Jamovi version 2.6.44.0 and RStudio version 4.3.3.

Besides standard statistical analyses, a control-relative concentration index was calculated to give an overall measure of compositional response. The index was defined as the average standardized (z-score) deviation from the respective irrigation-specific control for selected traits (dry matter, soluble solids, vitamin C, and titratable acidity). Positive values show higher concentrations compared to the control, while negative values show lower concentrations.

## 3 Rеsults

### 3.1 Effects of irrigation regime and biostimulant treatment on cumulative yield and averaged yield components

Seasonal cumulative yield showed a significant irrigation × treatment interaction (**Table 1**), indicating that treatment effects depended on the irrigation environment. However, the main effect of irrigation on total yield was not statistically significant. In contrast, yield components responded more clearly to irrigation environment, with fruit number per plant significantly increasing and average fruit weight significantly decreasing under reduced irrigation environment.

**Table 1:** General Linear Model (GLM) assessing the effects of irrigation environment (A), biostimulant treatment (B), and their interaction (A × B) on total yield and averaged yield components.

Source of variation	Yield per plant			Fruit number			Average fruit weight		
	F	p	Sign.	F	p	Sign.	F	p	Sign.
Irrigation environment (A)	0.845	0.360	ns	176.436	0.000	***	308.727	0.000	***
Treatment (B)	10.680	0.000	***	4.216	0.001	**	1.504	0.184	ns
А × B	3.135	0.007	**	1.568	0.164	ns	1.553	0.168	ns

F-values derived from general linear models including irrigation environment, biostimulant treatment, and their interaction as fixed factors. Significance levels: *p ≤ 0.05, **p ≤ 0.01, ***p ≤ 0.001; ns, not significant.

**Figure 1:**
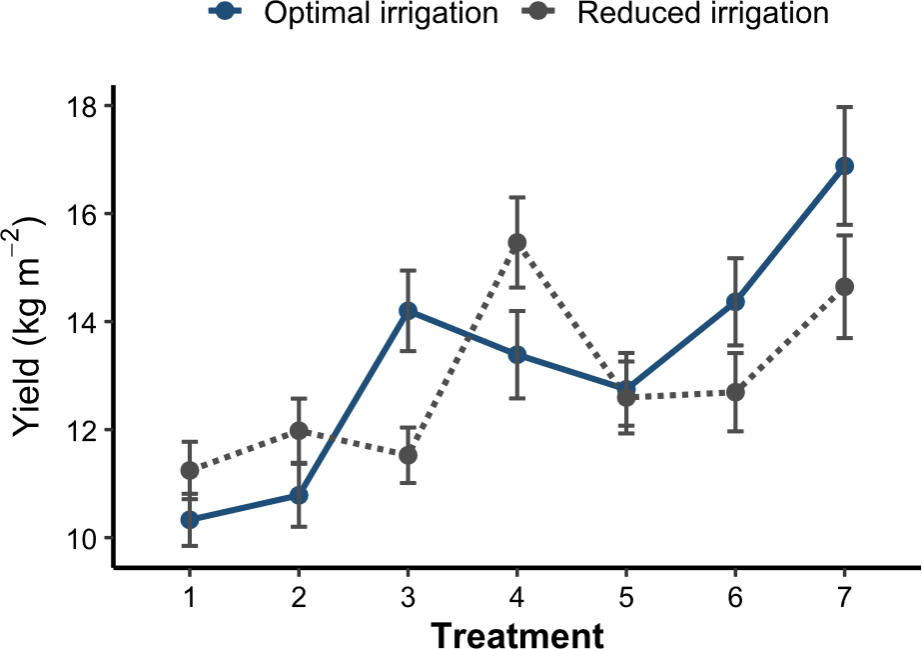
Interaction effect of irrigation regime and seaweed-based biostimulant treatments on total tomato yield expressed as kg m^−2^. Points represent means ± SE.

Under optimal irrigation, biostimulant treatments caused moderate variations in total yield, with several treatments showing higher values compared to the control (**Figure 1**). These differences were mainly linked to variations in fruit number, while average fruit weight stayed relatively consistent across treatments (**Table 2**). Overall, the effects of treatments under optimal irrigation were limited and did not significantly change the structure of yield formation.

**Table 2:** Effect of treatment with *Laminaria digitata (L.d)*, *Ascophyllum nodosum (A.n)* and their combination on yield components of tomatoes under optimal and reduced irrigation conditions.

Variant	Average number of fruits per plant		Average fruit weight, g	
Optimal irrigation				
1. Control	14.4	f	244.9	a
2. *L. digitata* – 0.7%	16.3	f	223.6	a
3. *L. digitata* – 1.0%	19.7	ef	243.4	a
4. *A. nodosum* – 0.7%	19.2	ef	232.8	a
5. *A. nodosum* – 1.0%	19.7	ef	218.0	a
6. *L.d + A.n* – 0.7%	20.7	def	232.6	a
7. *L.d + A.n* – 1.0%	21.9	cdef	260.2	a
Water deficit				
1. Control	28.9	abc	131.0	b
2. *L. digitata* – 0.7%	27.9	abcd	146.4	b
3. *L. digitata* – 1.0%	26.3	bde	148.6	b
4. *A. nodosum* – 0.7%	32.8	ab	160.6	b
5. *A. nodosum* – 1.0%	31.3	ab	136.7	b
6*. L.d + A.n* – 0.7%	29.0	abc	146.1	b
7. *L.d + A.n* – 1.0%	34.0	a	145.2	b

Within each column, means followed by different letters indicate statistically significant differences according to Tukey’s HSD test (p ≤ 0.05).

Under reduced irrigation environment, the distribution of yield components differed markedly from that observed under optimal conditions. Fruit number per plant was consistently higher, while average fruit weight was reduced across all treatments (**Table 2**), indicating a shift in yield structure under lower soil moisture. Differences among biostimulant treatments were present but generally moderate, and no single yield component alone explained the variation in total yield. This suggests that, under reduced irrigation environment, yield responses resulted from relatively small, combined changes across multiple components rather than from a dominant effect on either fruit number or fruit weight.

Taken together, these results indicate that the irrigation regime was the primary factor affecting yield structure, while biostimulant treatments caused secondary, context-dependent changes. The different responses of fruit number and fruit weight under reduced irrigation environment highlight the importance of evaluating yield components separately when analyzing total yield variations under different water availability conditions.

### 3.2 Temporal dynamics of yield formation under contrasting irrigation regimes

**Figure 2:**
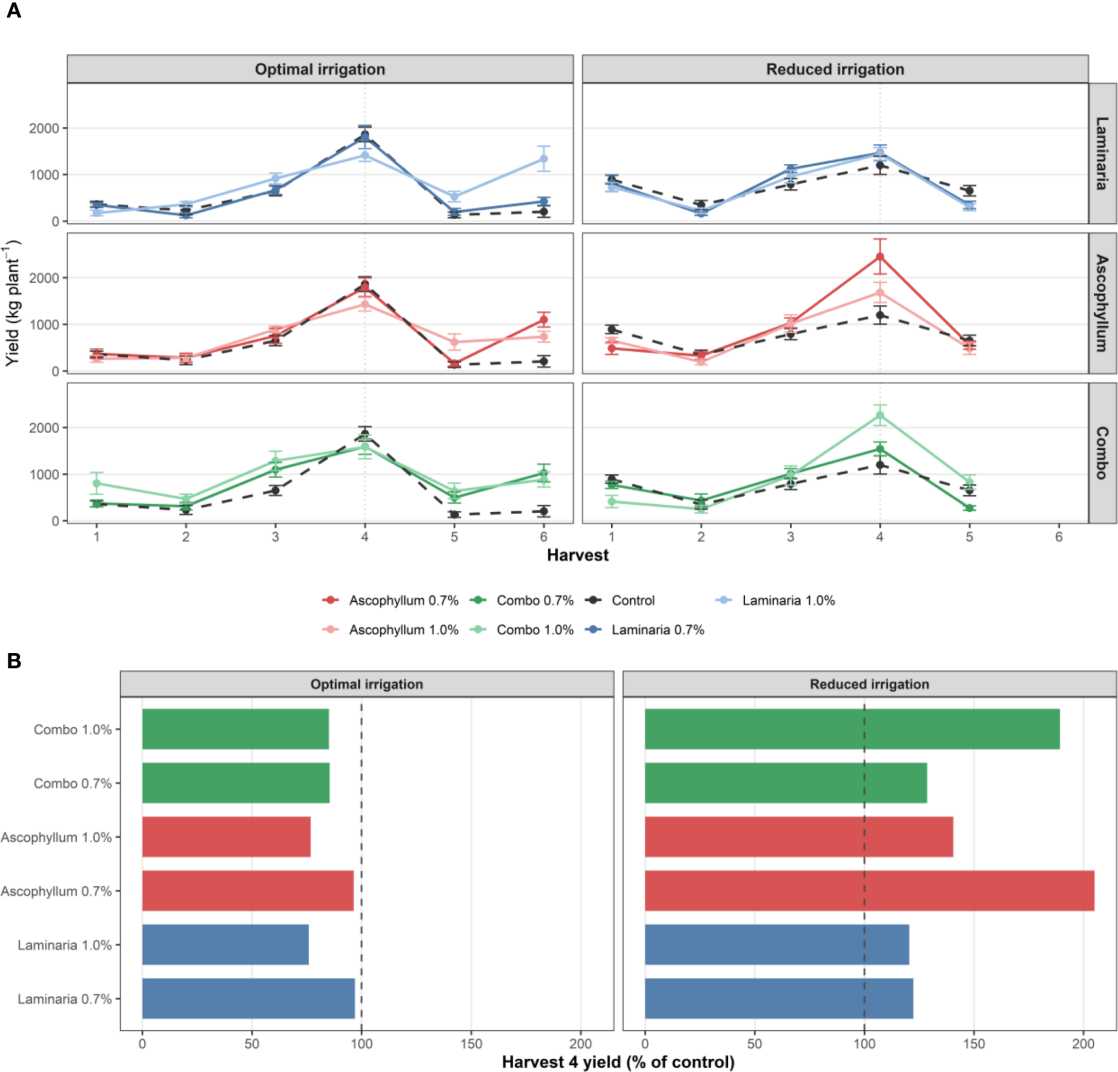
Biostimulant-induced restructuring of temporal yield allocation under contrasting irrigation regimes. Yield is expressed as kg plant^−1^. Panels display product-specific responses relative to the irrigation-specific control (dashed line). Harvest 4 represents the principal production peak. Values are means ± SE. **(A)** Peak yield response (harvest 4) expressed relative to the irrigation-specific control under optimal and reduced irrigation. Harvest 4 yield expressed as percentage of the respective irrigation control (100% dashed line).

Yield formation followed a structured temporal pattern that varied between irrigation regimes (**Figure 2A**). Under optimal irrigation, production was spread over six harvests, with a consistent peak at harvest 4 across all treatments. Variations among treatments were minimal, and differences mainly involved slight shifts in yield distribution before and after the peak rather than changes in peak intensity.

Under reduced irrigation environment, the temporal pattern of yield formation was more variable among treatments. Although harvest 4 remained the main production phase, the peak yield magnitude varied by treatment. When expressed relative to the irrigation-specific control (**Figure 2B**), several treatments showed higher relative values at the peak harvest, while others remained close to control levels. In contrast to optimal irrigation, where peak yield was relatively uniform, reduced irrigation conditions were associated with greater differentiation in peak expression.

Treatment effects under reduced irrigation environment were not evenly spread across the production cycle. Early harvests (H1–H2) showed relatively small differences among treatments, while later harvests tended to approach control values. The most significant differences appeared during the main production peak, suggesting that treatment effects were focused at specific times rather than consistently expressed throughout the growing period.

**Figure 3:**
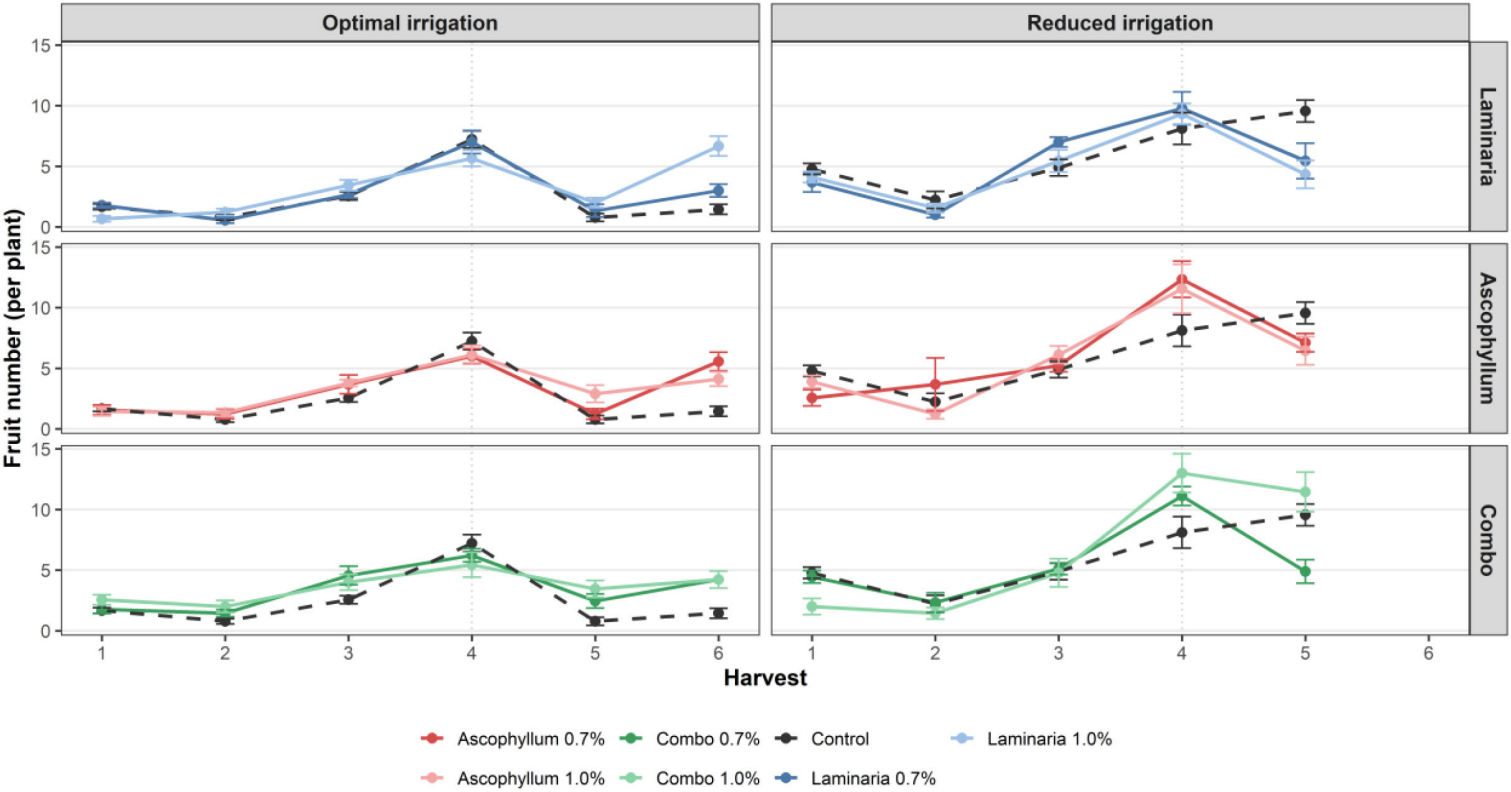
Treatment-dependent modulation of fruit number across harvests under contrasting irrigation regimes. Fruit number per plant recorded at each harvest. Panels represent *Laminaria, Ascophyllum*, and combined treatments relative to the irrigation-specific control (dashed line). The dotted vertical line indicates harvest 4 (principal production peak). Values are means ± SE.

The temporal dynamics of fruit number followed patterns similar to those observed for yield (**Figure 3**). Under optimal irrigation, the number of fruits at peak harvest was similar across treatments, indicating limited effects of treatments on reproductive intensity. Under reduced irrigation environment, some treatments were linked to higher fruit numbers at harvest 4, aligning with the observed differences in peak yield.

These observations were supported by the general linear model analysis (**Supplementary Table 1**), which showed significant effects of irrigation environment and harvest timing on yield dynamics. Fruit number was particularly responsive to irrigation environment, indicating that temporal variation in yield under contrasting irrigation conditions was primarily associated with changes in fruit number rather than fruit size. Overall, the results suggest that under reduced irrigation environment, treatment effects were expressed mainly through modifications in the timing and intensity of yield formation rather than through consistent changes across the entire production period.

### 3.3 Fruit quality

Fruit compositional traits were mainly affected by the irrigation regime (**Supplementary Tables 2**–**S4**). Across all treatments, reduced irrigation was associated with higher levels of dry matter, soluble solids, vitamin C, and titratable acidity (**Supplementary Table 3**), indicating consistent changes in fruit composition under lower soil moisture conditions.

Increased dry matter, soluble solids, and titratable acidity may partly reflect reduced dilution within the fruit tissues, whereas changes in vitamin C and ionic composition may additionally involve stress-related metabolic and physiological responses. Therefore, the variations in compositional traits should be understood as stemming from both concentration-related processes and physiological responses to decreased water availability, rather than as uniform improvements in fruit quality.

Compared to irrigation effects, the influence of biostimulant treatments on compositional traits was more selective and parameter-specific. Significant effects of treatment and of the treatment × irrigation interaction were identified for several variables (**Supplementary Table 2**), indicating that treatment responses depended on the irrigation regime. However, no consistent pattern of simultaneous increase across all compositional traits was observed within individual treatments (**Supplementary Table 3**).

**Figure 4:**
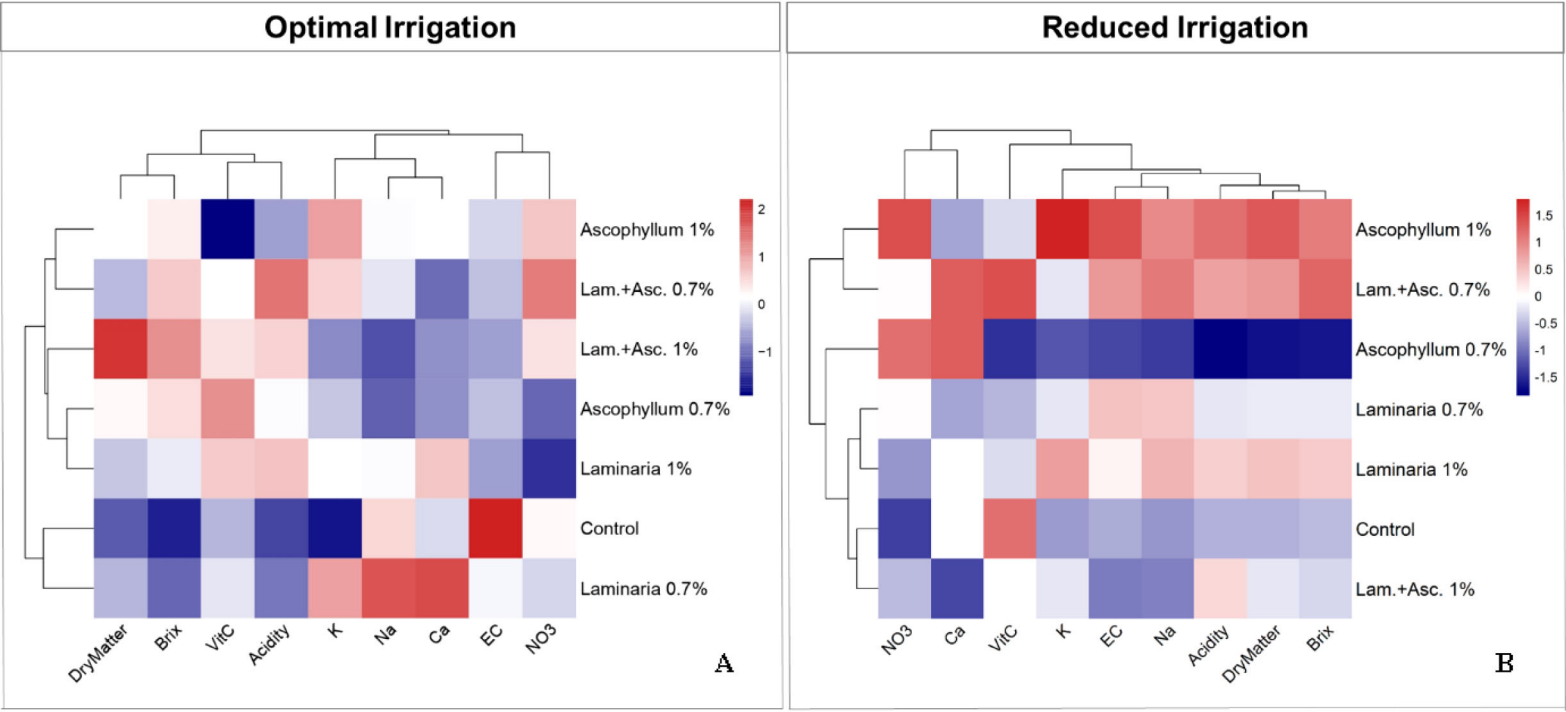
Hierarchical clustering heatmaps of standardized fruit compositional and mineral parameters under **(A)** optimal and **(B)** reduced irrigation.

To further examine treatment-related compositional patterns, hierarchical clustering of standardized biochemical and ionic variables was performed (**Figure 4**). Under optimal irrigation, treatments showed relatively limited separation, with clustering primarily reflecting differences among variables rather than clear treatment grouping (**Figure 4A**). In contrast, under reduced irrigation environment, treatment differentiation became more pronounced, with clustering patterns more strongly influenced by ionic variables (**Figure 4B**). This indicates that compositional responses were more heterogeneous under reduced irrigation and that treatment effects were more distinctly expressed under lower water availability.

Ionic composition responded to both irrigation regime and treatment (**Supplementary Table 4**). Under reduced irrigation environment, variability among treatments was more pronounced, particularly for Na^+^ and Ca²^+^ concentrations, indicating treatment-dependent differences in ion balance under varying water availability. However, the direction and extent of these responses varied among treatments and did not follow a consistent pattern.

In contrast to compositional traits, fruit firmness and color parameters showed limited variation among treatments and irrigation regimes (**Supplementary Table 5**). The absence of consistent differences in these attributes indicates that structural and visual fruit characteristics remained relatively stable under the experimental conditions.

In summary, fruit compositional responses were predominantly driven by irrigation regime, while biostimulant treatments introduced additional variability that depended on both the specific parameter and the irrigation environment. These results indicate that compositional changes under reduced irrigation environment reflect a combination of irrigation-induced effects and treatment-specific responses rather than a uniform enhancement of fruit quality.

### 3.4 Integrative analysis of yield and compositional traits

To assess the combined variation in productivity and fruit compositional traits, principal component analysis (PCA) was performed using yield, fruit number, biochemical, and mineral parameters. The first two principal components explained 53.3% and 21.4% of the total variance, respectively, together accounting for 74.7% of the variability in the dataset (**Supplementary Table 6**; **Supplementary Figure 2**).

**Figure 5:**
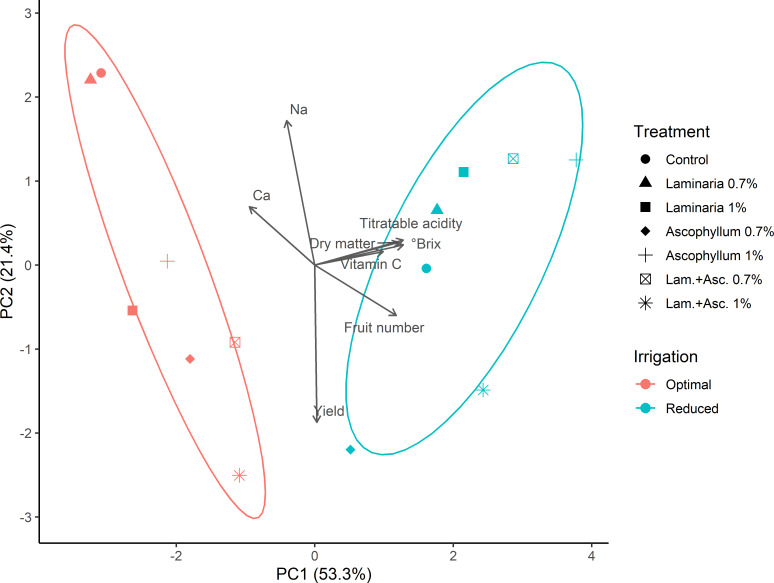
Principal component analysis (PCA) integrating yield, fruit number, compositional and mineral traits under optimal and reduced irrigation.

The first principal component (PC1) primarily separated the irrigation regimes, with reduced irrigation associated with higher values of dry matter, soluble solids, vitamin C, and titratable acidity (**Figure 5**). These variables showed strong positive loadings on PC1 (**Supplementary Table 6**), indicating that compositional variation was mainly driven by irrigation-related effects. In contrast, yield contributed only a minor amount to PC1 and was more strongly associated with PC2 (**Supplementary Table 6**; **Figure 5**), suggesting that variation in productivity was partly independent of the main compositional gradient. Detailed variable loadings and percentage contributions to PC1 and PC2 are provided in **Supplementary Table 6**, and the scree plot is shown in **Supplementary Figure 1**.

The second principal component (PC2) was primarily influenced by yield, along with Na^+^ and EC (**Supplementary Table 6**; **Figure 5**), indicating additional differences among treatments along a productivity–ionic composition axis. Within the reduced irrigation group, treatments showed different positions in the multivariate space, highlighting variability in the combined expression of yield and compositional traits. However, these differences did not correspond to a consistent directional shift across all variables.

**Figure 6:**
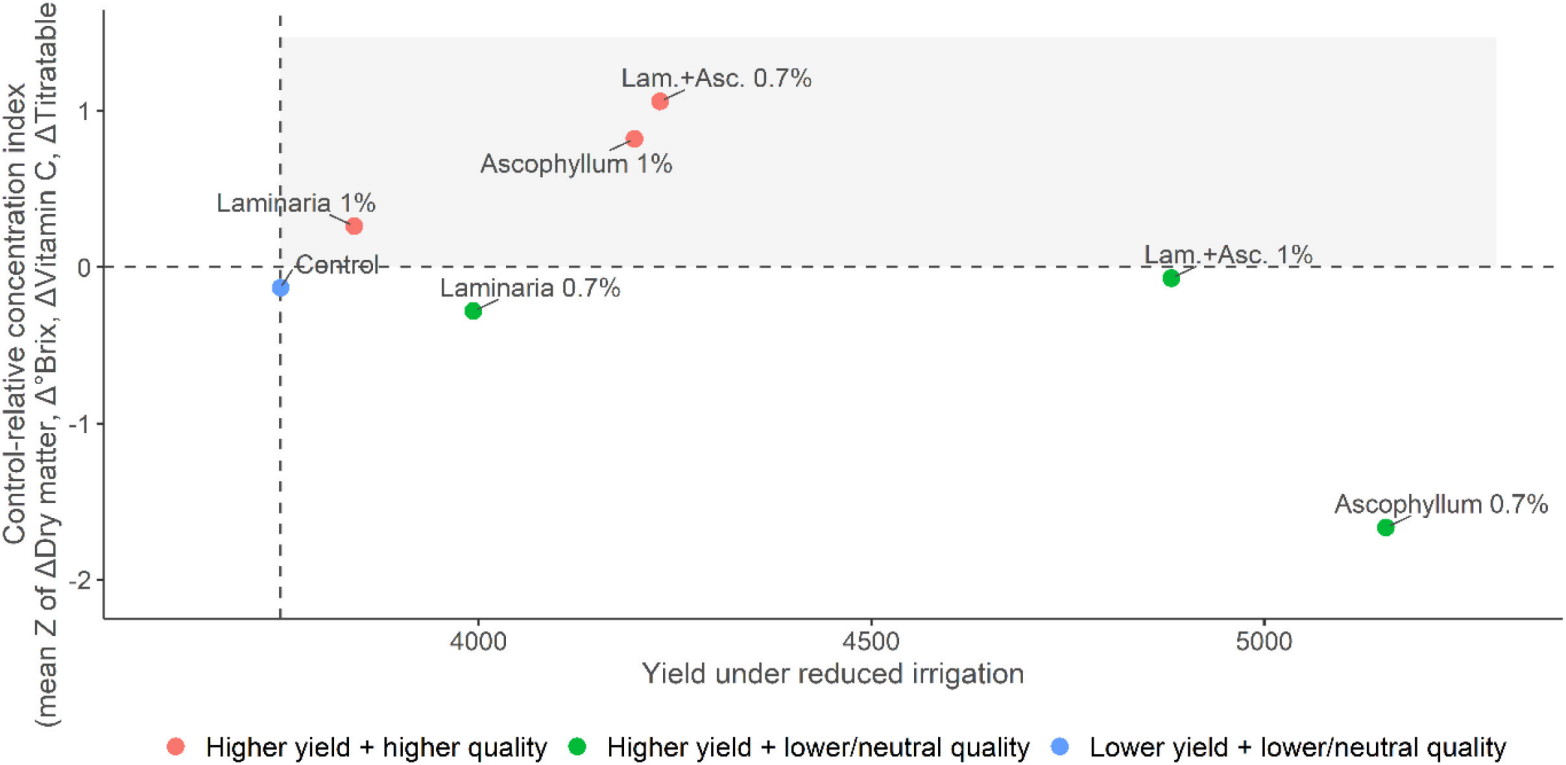
Relationship between yield and control-relative concentration index under reduced irrigation. The concentration index was calculated as the mean standardized (z-score) difference relative to the control for dry matter, °Brix, vitamin C, and titratable acidity. The vertical dashed line indicates the yield of the control under reduced irrigation, while the horizontal dashed line represents no compositional change relative to the control (Δ-index = 0). The shaded upper-right quadrant highlights treatments exhibiting both higher yield and increased compositional concentration relative to the control.

Along with PCA, relative yield performance under reduced irrigation environment was expressed as a percentage of the irrigation-specific control (**Figure 6**). This index indicated that some treatments had higher relative yields than the control, while others remained close to control levels. Differences among treatments were less noticeable under optimal irrigation (data not shown).

The relationship between yield and the control-relative concentration index under reduced irrigation (**Figure 6**) indicated that compositional trait increases were not always matched by proportional increases in yield. Treatments with higher concentration index values did not necessarily yield higher productivity, whereas moderate compositional changes were often associated with relatively stable yield levels. This pattern indicates that compositional variation and yield responses were only partly connected under reduced irrigation conditions.

The integrative analysis supports the interpretation that irrigation regime was the primary driver of compositional variation, while yield responses and treatment effects were expressed along a partially independent axis. These results highlight that changes in fruit composition and productivity under reduced irrigation environment should be interpreted as related but not directly proportional processes.

The concentration index was calculated as the mean standardized (z-score) difference relative to the control for dry matter, °Brix, vitamin C, and titratable acidity. The vertical dashed line indicates the yield of the control under reduced irrigation, while the horizontal dashed line represents no compositional change relative to the control (Δ-index = 0). The shaded upper-right quadrant highlights treatments exhibiting both higher yield and increased compositional concentration relative to the control.

## 4 Discussion

### 4.1 Context-dependent effects of biostimulants under contrasting irrigation regimes

Plant biostimulants are generally characterized by their effects on plant growth, such as improving nutrient efficiency and helping plants cope with less-than-ideal environmental conditions (du Jardin, 2015; Calvo et al., 2014; Colla and Rouphael, 2015; Rouphael and Colla, 2020; Rabhi et al., 2025). However, their success often depends heavily on the growing environment, plant species, and management practices.

In the current study, treatment effects were closely linked with the irrigation regime. With optimal watering, biostimulant use caused only minor differences in yield and fruit quality, showing that under sufficient water conditions, treatment effects were relatively small. Conversely, under reduced watering, greater variation among treatments was observed, particularly in yield development during the main growing phase. These results suggest that treatment effects were mainly evident under conditions of reduced water availability, but should be understood within the context of specific irrigation environments rather than as clear evidence of improved stress tolerance. Since plant physiological parameters such as water potential, photosynthesis, or hormonal responses were not measured, the responses observed probably indicate treatment-related adjustments in plant performance under lower soil moisture rather than a confirmed mechanistic stress-mitigation effect.

Previous studies have indicated that seaweed-derived products, especially those based on *Ascophyllum nodosum*, can affect how plants respond to water stress, although the extent and type of these effects depend on extract composition, concentration, and application methods (Shukla et al., 2019; Carmody et al., 2020; Ali et al., 2021; Espinosa-Antón et al., 2025). The current results align with this context-dependent behavior, emphasizing that treatment effects may change based on water availability and crop management practices (Goñi et al., 2018; Ahmed et al., 2022).

### 4.2 Temporal modulation of yield formation under reduced irrigation

One of the main findings of this study is that differences in yield under reduced irrigation environment were not consistent throughout the entire production cycle but were mainly observed during the peak harvest period. This pattern suggests that treatment effects were time-specific, influencing the distribution of yield rather than causing a uniform increase across all harvests.

The increase in yield observed in some treatments under reduced irrigation was mainly due to higher fruit number, while average fruit weight remained relatively stable. This suggests that treatment effects were more closely related to processes influencing fruit set or retention during critical developmental stages rather than to changes in fruit growth.

Tomato fruit development is closely linked to source–sink relationships, and fruit set is sensitive to changes in assimilate availability and environmental conditions (Kang et al., 2011; Osorio et al., 2014). Under water-limited conditions, variations in carbon partitioning and plant growth patterns may influence reproductive processes, especially during critical developmental stages.

In the present study, no direct measurements of assimilate supply, flower dynamics, or plant physiological status were performed. Therefore, the observed increase in fruit number at the peak harvest should be interpreted as an indication of treatment-associated differences in reproductive performance under reduced irrigation, rather than as direct evidence of specific physiological mechanisms.

The results from our study highlight that, under reduced irrigation environment, treatment effects were expressed primarily through changes in the timing and intensity of yield formation, emphasizing the importance of accounting for temporal yield dynamics when evaluating treatment performance under contrasting water availability conditions.

### 4.3 Irrigation-driven concentration effects and biochemical adjustments

Across treatments, reduced irrigation consistently related to higher levels of dry matter, soluble solids, vitamin C, and titratable acidity. These findings agree with previous studies showing that water deficit can lead to increased concentrations of soluble metabolites and organic acids in tomato fruits (Patanè and Cosentino, 2010; Ripoll et al., 2014, 2016; Lovelli et al., 2017). However, such increases should be viewed with caution, as they may result from a combination of reduced fruit water content (concentration effect) and changes in metabolic activity under water-limited conditions (Guichard et al., 2001; Ripoll et al., 2014). In this context, higher concentrations of compositional traits do not necessarily indicate improved physiological performance or better fruit quality in a broader agronomic sense.

In the present study, the irrigation regime was the main factor influencing compositional variation, as confirmed by both univariate analysis (**Supplementary Table 2**; **Supplementary Table 3**) and multivariate patterns (**Figure 5**). In contrast, the effects of biostimulant treatments were more selective and specific to certain parameters, with significant interactions seen for some variables but without a consistent trend of increasing across all traits.

Particularly, treatment effects were more noticeable in the ionic composition, especially for Na^+^ and Ca²^+^, indicating that some treatments affected ion balance under different irrigation conditions. However, these changes did not result in a consistent compositional response across biochemical parameters, showing that treatment effects were not uniformly expressed across various aspects of fruit composition.

### 4.4 Yield–quality decoupling and trade-off thresholds

The integrative analysis of yield and compositional traits indicated that these two components did not respond in a fully coordinated manner under reduced irrigation. While concentration-related traits (dry matter, soluble solids, vitamin C, and titratable acidity) increased consistently with reduced water availability, yield responses varied among treatments and were primarily associated with fruit number rather than compositional changes.

This pattern aligns with previous findings showing that moderate water deficit can boost concentration-related traits while keeping yield within a certain range. However, more severe constraints might cause a divergence between productivity and composition depending on the stress level and developmental stage (Ripoll et al., 2014, 2016; Patanè and Cosentino, 2010).

In the present study, the relationship between yield and the control-relative concentration index (**Figure 6**) indicated that treatments with higher compositional values did not consistently exhibit higher yields. Conversely, treatments associated with moderate compositional changes were often linked to relatively stable productivity. This suggests that compositional enhancement and yield formation were only partly coupled under reduced irrigation conditions.

The PCA results (**Figure 5**; **Supplementary Table 6**) further supported this interpretation, showing that yield and concentration-related variables were associated with different principal components, indicating partially independent axes of variation. However, these patterns should be interpreted as indicating coordinated, not directly proportional, responses rather than as evidence of a strict trade-off mechanism.

### 4.5 Implications for greenhouse tomato management

From a greenhouse management perspective, the results show that irrigation regime plays a key role in influencing both yield formation and fruit compositional traits. The observed changes in yield structure and compositional parameters under reduced irrigation align with previous studies emphasizing the importance of irrigation management for balancing productivity and fruit quality in tomato (Patanè and Cosentino, 2010; Ripoll et al., 2014; Lovelli et al., 2017).

The findings also indicate that biostimulant treatments might affect crop performance under reduced irrigation, especially in the timing of yield and fruit number. Nonetheless, these effects were specific to each treatment and depended on the irrigation conditions, and they did not consistently lead to improvements across all compositional traits. This aligns with previous studies showing that biostimulant effectiveness is heavily influenced by environmental conditions and management practices (Calvo et al., 2014; Rouphael and Colla, 2020).

Therefore, using biostimulants under reduced irrigation should be part of a broader management strategy that combines irrigation scheduling, crop growth stages, and production goals. Instead of solely boosting yield or quality, biostimulant application may help change how yield is spread over time and how compositional traits respond to water availability.

### 4.6 Limitations and future research perspectives

Several limitations of the current study should be acknowledged. First, the irrigation regimes were implemented in separate greenhouse compartments due to technical constraints of the experimental facility. Consequently, the irrigation factor lacked independent compartment-level replication and was partially confounded with compartment-related microclimatic differences, including air temperature and relative humidity. Nevertheless, such coupled soil-atmosphere responses are commonly associated with irrigation management in greenhouse systems and may also reflect practical production conditions. Therefore, irrigation-related effects should be interpreted as differences between practical greenhouse irrigation environments rather than as fully replicated irrigation treatment effects. Although the compartments followed the same agronomic practices, plant density, fertilization, and crop management protocols, compartment-associated environmental effects cannot be fully separated from irrigation effects in the present design.

Second, plant physiological parameters such as water potential, gas exchange, or hormonal responses were not measured. As a result, the study does not provide direct evidence of the physiological mechanisms underlying the observed treatment responses. The interpretation of treatment effects is therefore based on yield components and fruit compositional traits, which represent integrated outcomes of plant performance.

Third, although soil moisture was continuously monitored and kept within clearly defined ranges for different irrigation regimes, plant-available water was not measured using soil water potential measurements. As a result, the level of water limitation can be described in relative terms but cannot be fully understood in physiological terms. In addition, field capacity and permanent wilting point were not determined for the experimental substrate, which further limits the physiological interpretation of stress intensity.

Finally, the experiment was carried out in a single greenhouse environment using one tomato genotype. Since responses to both irrigation and biostimulant treatments may differ depending on genotype and environmental conditions (Ripoll et al., 2016), additional studies involving different cultivars and growing conditions would be needed to verify the broader applicability of the observed patterns.

Future research combining physiological measurements, such as plant water status, photosynthetic activity, and hormonal regulation, with temporal yield analysis would provide deeper insight into the mechanisms underlying treatment responses. In addition, multi-environment and multi-season experiments would help to validate the robustness of the observed relationships between irrigation, yield formation, and fruit composition.

## 5 Conclusions

This study demonstrates that irrigation regime is the primary factor shaping yield formation and fruit compositional traits in greenhouse-grown tomato. Reduced irrigation consistently altered yield structure by increasing fruit number and decreasing average fruit weight, while also increasing concentrations of key compositional parameters, including dry matter, soluble solids, vitamin C, and titratable acidity.

The results further show that yield and compositional responses under reduced irrigation were only partly coupled. While compositional traits increased consistently with reduced water availability, yield responses varied among treatments and were mainly associated with fruit number rather than compositional changes. This indicates that productivity and fruit composition followed related but not directly proportional response patterns.

Temporal yield analysis further indicated that treatment effects under reduced irrigation were expressed mainly through modifications in the timing and intensity of peak production rather than through uniform effects across the entire production cycle. The integrative PCA and concentration-index approach additionally showed that compositional enhancement was not necessarily associated with proportional increases in productivity.

Biostimulant treatments affected crop responses depending on the context, with stronger effects seen under reduced irrigation. However, these effects were not consistent across all parameters and did not always lead to simultaneous increases in yield and compositional traits.

Overall, the findings highlight that crop responses under reduced irrigation should be interpreted as the combined outcome of irrigation-driven effects and treatment-specific variability. From a management perspective, irrigation remains the dominant factor determining crop performance, while additional inputs, such as seaweeds biostimulants, may modify yield distribution and compositional responses under specific conditions.

## Data Availability

The raw data supporting the conclusions of this article will be made available by the authors, without undue reservation.
