# Supplementary material for: Yield-quality relationships under deficit irrigation in greenhouse tomato: modulation by seaweed biostimulants
Source: Front Plant Sci. 2026 May 26;17:1859338. doi: 10.3389/fpls.2026.1859338 (PMC13246414; doi:10.3389/fpls.2026.1859338)
Supplement: Supplementary file 1 [file SupplementaryFile1.docx]

**Supplementary tables and figures**

**Table S1.** General linear model (GLM) assessing the effects of irrigation environment, biostimulant treatment and harvest timing on yield dynamics in tomato

|  | Yield per plant | | | Fruit number | | | Average fruit weight | | |
| --- | --- | --- | --- | --- | --- | --- | --- | --- | --- |
|  | F | p | Sign. | F | p | Sign. | F | p | Sign. |
| Irrigation environment | 8.995 | 0.003 | ** | 178.662 | 0.000 | *** | 86.901 | 0.000 | *** |
| Treatment | 4.541 | 0.000 | *** | 1.845 | 0.088 | ns | 3.970 | 0.001 | ** |
| Harvest | 153.806 | 0.000 | *** | 101.007 | 0.000 | *** | 29.782 | 0.000 | *** |

F-values derived from general linear models including irrigation environment, biostimulant treatment and harvest timing as fixed factors. Significance levels: * p ≤ 0.05, ** p ≤ 0.01, *** p ≤ 0.001; ns – not significant

**Table S2.** General linear model (GLM) assessing the effects of irrigation environment, biostimulant treatment, and their interaction on fruit quality parameters in tomato

| Parameter | Irrigation environment | | | Treatment | | | Irrigation × Treatment | | |
| --- | --- | --- | --- | --- | --- | --- | --- | --- | --- |
|  | F | p | Sign. | F | p | Sign. | F | p | Sign. |
| Dry matter | 56.352 | 0.000 | *** | 2.487 | 0.047 | * | 2.897 | 0.025 | * |
| °Brix | 178.314 | 0.000 | *** | 4.070 | 0.005 | ** | 3.166 | 0.017 | * |
| Vitamin C | 72.670 | 0.000 | *** | 4.064 | 0.005 | ** | 7.165 | 0.000 | *** |
| pH | 29.209 | 0.000 | *** | 3.607 | 0.009 | ** | 1.922 | 0.112 | ns |
| Titratable acidity | 237.778 | 0.000 | *** | 4.721 | 0.002 | ** | 3.313 | 0.014 | * |
| NO₃⁻ | 14.080 | 0.001 | ** | 2.916 | 0.025 | * | 2.548 | 0.043 | * |
| Na⁺ | 6.674 | 0.015 | * | 6.479 | 0.000 | *** | 2.039 | 0.093 | ns |
| Ca²⁺ | 26.042 | 0.000 | *** | 2.903 | 0.025 | * | 5.625 | 0.001 | ** |
| K⁺ | 34.031 | 0.000 | *** | 3.021 | 0.021 | ** | 0.854 | 0.540 | ns |
| EC | 11.368 | 0.002 | ** | 8.930 | 0.000 | *** | 11.310 | 0.000 | *** |
| Firmness | 0.000 | 0.984 | ns | 1.415 | 0.244 | ns | 0.754 | 0.611 | ns |
| L* | 5.736 | 0.024 | * | 2.476 | 0.048 | * | 0.766 | 0.603 | ns |
| a* | 20.284 | 0.000 | *** | 2.087 | 0.087 | ns | 1.115 | 0.379 | ns |
| b* | 17.146 | 0.000 | *** | 2.772 | 0.030 | * | 1.362 | 0.264 | ns |
| Chroma | 23.774 | 0.000 | *** | 2.733 | 0.032 | * | 1.380 | 0.257 | ns |
| Hue | 0.018 | 0.893 | ns | 0.661 | 0.681 | ns | 0.479 | 0.818 | ns |

F-values derived from general linear models including irrigation environment, biostimulant treatment, and their interaction as fixed factors; *, **, *** - significance at p ≤ 0.05, p ≤ 0.01 and p ≤ 0.001 respectively; ns – non significant difference

**Table S3.** Biochemical fruit quality traits under optimal and reduced irrigation

| **Variant** | | **Vit. C, mg/100g FW** | | **Dry matter, %** | | **Total soluble solids, ^о^Brix** | | **pH** | | **Titratable organic acids, % citric acid** | |
| --- | --- | --- | --- | --- | --- | --- | --- | --- | --- | --- | --- |
| Optimal irrigation | 1 | 18.7 | de | 5.2 | d | 4.4 | e | 4.55 | a | 0.38 | d |
|  | 2 | 19.2 | c-e | 5.4 | d | 4.5 | e | 4.55 | a | 0.38 | d |
|  | 3 | 20.3 | c-e | 5.5 | d | 4.7 | e | 4.53 | ab | 0.41 | d |
|  | 4 | 21.1 | cd | 5.7 | cd | 4.8 | e | 4.52 | abc | 0.39 | d |
|  | 5 | 16.5 | e | 5.7 | d | 4.8 | e | 4.52 | abc | 0.39 | d |
|  | 6 | 19.5 | c-e | 5.5 | d | 4.8 | de | 4.49 | abc | 0.41 | cd |
|  | 7 | 20.0 | c-e | 6.4 | bcd | 4.9 | c-e | 4.48 | abc | 0.40 | d |
| Water deficit | 1 | 25.1 | ab | 6.5 | abcd | 5.6 | abcd | 4.41 | bc | 0.48 | abc |
|  | 2 | 21.6 | bcd | 6.8 | abcd | 5.8 | ab | 4.49 | abc | 0.50 | ab |
|  | 3 | 22.1 | abcd | 7.2 | abc | 6.0 | ab | 4.41 | bc | 0.52 | a |
|  | 4 | 19.7 | cde | 5.8 | cd | 5.2 | bde | 4.51 | abc | 0.44 | bcd |
|  | 5 | 22.1 | abcd | 7.8 | a | 6.2 | a | 4.51 | abc | 0.54 | a |
|  | 6 | 25.6 | a | 7.5 | ab | 6.3 | a | 4.41 | bc | 0.53 | a |
|  | 7 | 22.7 | abc | 6.8 | abcd | 5.7 | abc | 4.40 | c | 0.51 | a |

a,b,c - in each column, different letters indicate a statistically significant difference according to Tukey's HSD test, p≤0.05

**Table S4.** Ionic composition and electrical conductivity of tomato fruits under optimal and reduced irrigation

| **Variant** | | **NO₃⁻, ppm** | | **Na⁺, ppm** | | **Ca²⁺, ppm** | | **K⁺, ppm** | | **ЕС, mS/cm** | |
| --- | --- | --- | --- | --- | --- | --- | --- | --- | --- | --- | --- |
| Optimal irrigation | 1 | 163.3 | abc | 34.3 | ab | 5.7 | bc | 1666.7 | d | 5.1 | a |
|  | 2 | 156.7 | abc | 37.0 | a | 8.0 | a | 1866.7 | abd | 4.3 | bcd |
|  | 3 | 136.7 | c | 33.3 | abc | 6.7 | ab | 1800.0 | bcd | 4.1 | d |
|  | 4 | 143.3 | bc | 30.7 | bc | 5.0 | bc | 1766.7 | bcd | 4.2 | cd |
|  | 5 | 170.0 | abc | 33.3 | abc | 6.0 | a-c | 1866.7 | abcd | 4.2 | cd |
|  | 6 | 180.0 | ab | 33.0 | abc | 4.7 | bc | 1833.3 | abcd | 4.2 | cd |
|  | 7 | 166.7 | abc | 30.3 | bc | 5.0 | bc | 1733.3 | cd | 4.1 | d |
| Water deficit | 1 | 160.0 | abc | 30.0 | bc | 4.7 | bc | 1900.0 | abcd | 4.4 | bcd |
|  | 2 | 176.7 | abc | 32.7 | abc | 4.3 | c | 1933.3 | abc | 4.6 | bc |
|  | 3 | 166.7 | abc | 33.0 | abc | 4.7 | bc | 2000.0 | ab | 4.5 | bcd |
|  | 4 | 190.0 | a | 28.7 | c | 5.3 | bc | 1866.7 | abcd | 4.3 | bcd |
|  | 5 | 193.3 | a | 33.7 | abc | 4.3 | c | 2066.7 | a | 4.7 | ab |
|  | 6 | 176.7 | abc | 34.0 | abc | 5.3 | bc | 1933.3 | abc | 4.6 | bc |
|  | 7 | 170.0 | abc | 29.7 | bc | 4.0 | c | 1933.3 | abc | 4.3 | bcd |

a,b,c - in each column, different letters indicate a statistically significant difference according to Tukey's HSD test, p≤0.05

**Table S5.** Physical fruit traits under optimal and reduced irrigation

| **Variant** | | **Hardness, ^о^Shore** | | **L*** | | **a*** | | **b*** | | **Chroma** | | **Hue** | | |
| --- | --- | --- | --- | --- | --- | --- | --- | --- | --- | --- | --- | --- | --- | --- |
| Optimal irrigation | 1 | 19.4 | ns | 39.4 | ab | 21.9 | abc | 14.7 | ab | 26.4 | bc | 33.9 | ns |  |
|  | 2 | 20.3 | ns | 39.5 | ab | 22.2 | abc | 15.2 | ab | 27.0 | abc | 34.4 | ns |  |
|  | 3 | 20.2 | ns | 38.6 | b | 21.3 | c | 14.4 | b | 25.7 | c | 34.0 | ns |  |
|  | 4 | 22.9 | ns | 39.0 | b | 21.5 | bc | 13.9 | b | 25.6 | c | 32.9 | ns |  |
|  | 5 | 20.7 | ns | 39.7 | ab | 22.2 | abc | 14.9 | ab | 26.8 | abc | 33.9 | ns |  |
|  | 6 | 19.2 | ns | 40.2 | ab | 22.3 | abc | 14.8 | ab | 26.8 | abc | 33.6 | ns |  |
|  | 7 | 17.0 | ns | 39.8 | ab | 21.5 | bc | 15.3 | ab | 26.4 | bc | 35.4 | ns |  |
| Water deficit | 1 | 19.9 | ns | 39.6 | ab | 23.1 | abc | 15.4 | ab | 27.8 | abc | 33.8 | ns |  |
|  | 2 | 20.4 | ns | 39.7 | ab | 22.4 | abc | 15.5 | ab | 27.3 | abc | 34.8 | ns |  |
|  | 3 | 22.6 | ns | 39.9 | ab | 22.7 | abc | 15.0 | ab | 27.2 | abc | 33.5 | ns |  |
|  | 4 | 21.7 | ns | 39.6 | ab | 22.9 | abc | 15.1 | ab | 27.4 | abc | 33.5 | ns |  |
|  | 5 | 18.5 | ns | 40.8 | ab | 25.3 | ab | 17.2 | a | 30.6 | ab | 34.2 | ns |  |
|  | 6 | 16.5 | ns | 42.4 | a | 25.6 | a | 17.4 | a | 31.0 | a | 34.3 | ns |  |
|  | 7 | 20.2 | ns | 39.9 | ab | 23.8 | abc | 15.9 | ab | 28.6 | abc | 33.8 | ns |  |

a,b,c - in each column, different letters indicate a statistically significant difference according to Tukey's HSD test, p≤0.05; ns – non significant difference

**Table S6.** Loadings and percentage contribution of variables to the first two principal components (PC1 and PC2) in the integrative PCA of yield, compositional and mineral traits

| Variable | PC1 loading | PC2 loading | PC1 contribution | PC2 contribution | Total contribution |
| --- | --- | --- | --- | --- | --- |
| Yield | 0.010 | -0.592 | 0.0 | 35.0 | 35.0 |
| Na⁺ | -0.128 | 0.543 | 1.6 | 29.5 | 31.1 |
| EC | 0.096 | 0.474 | 0.9 | 22.4 | 23.4 |
| Fruit number | 0.372 | -0.190 | 13.9 | 3.6 | 17.5 |
| Acidity | 0.404 | 0.094 | 16.3 | 0.9 | 17.2 |
| Brix | 0.404 | 0.076 | 16.3 | 0.6 | 16.9 |
| Dry matter | 0.385 | 0.085 | 14.8 | 0.7 | 15.5 |
| Ca²⁺ | -0.298 | 0.219 | 8.9 | 4.8 | 13.7 |
| K⁺ | 0.338 | 0.148 | 11.4 | 2.2 | 13.6 |
| Vitamin C | 0.312 | 0.050 | 9.8 | 0.3 | 10.0 |
| NO₃⁻ | 0.247 | -0.006 | 6.1 | 0.0 | 6.1 |

Variables were centered and scaled prior to PCA. Contributions (%) were calculated from squared loadings


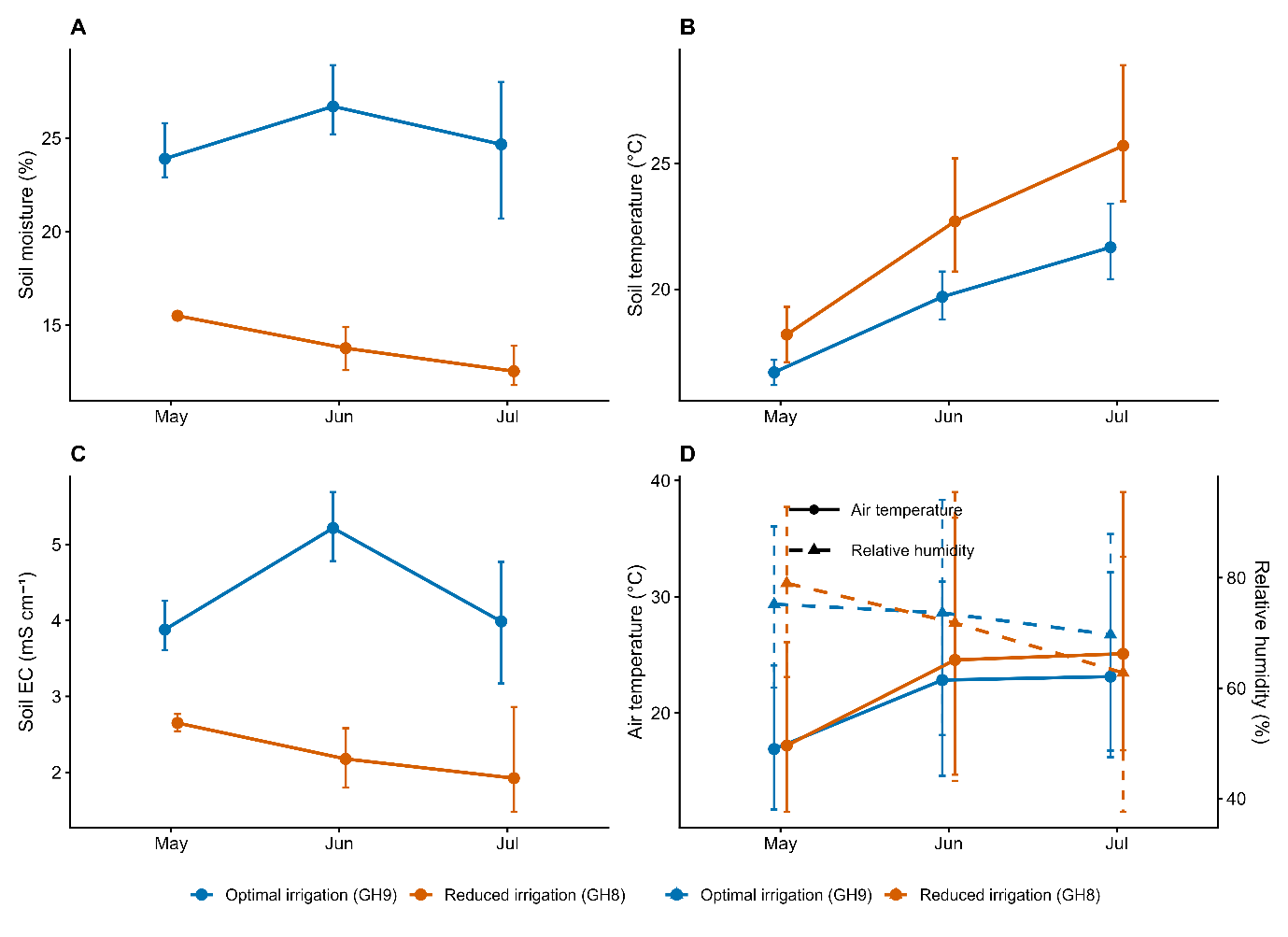


**Figure S1.** Seasonal dynamics of soil and air microclimatic parameters under optimal and reduced irrigation regimes in greenhouse compartments. Values are presented as monthly means, while error bars represent the minimum and maximum temperatures

**
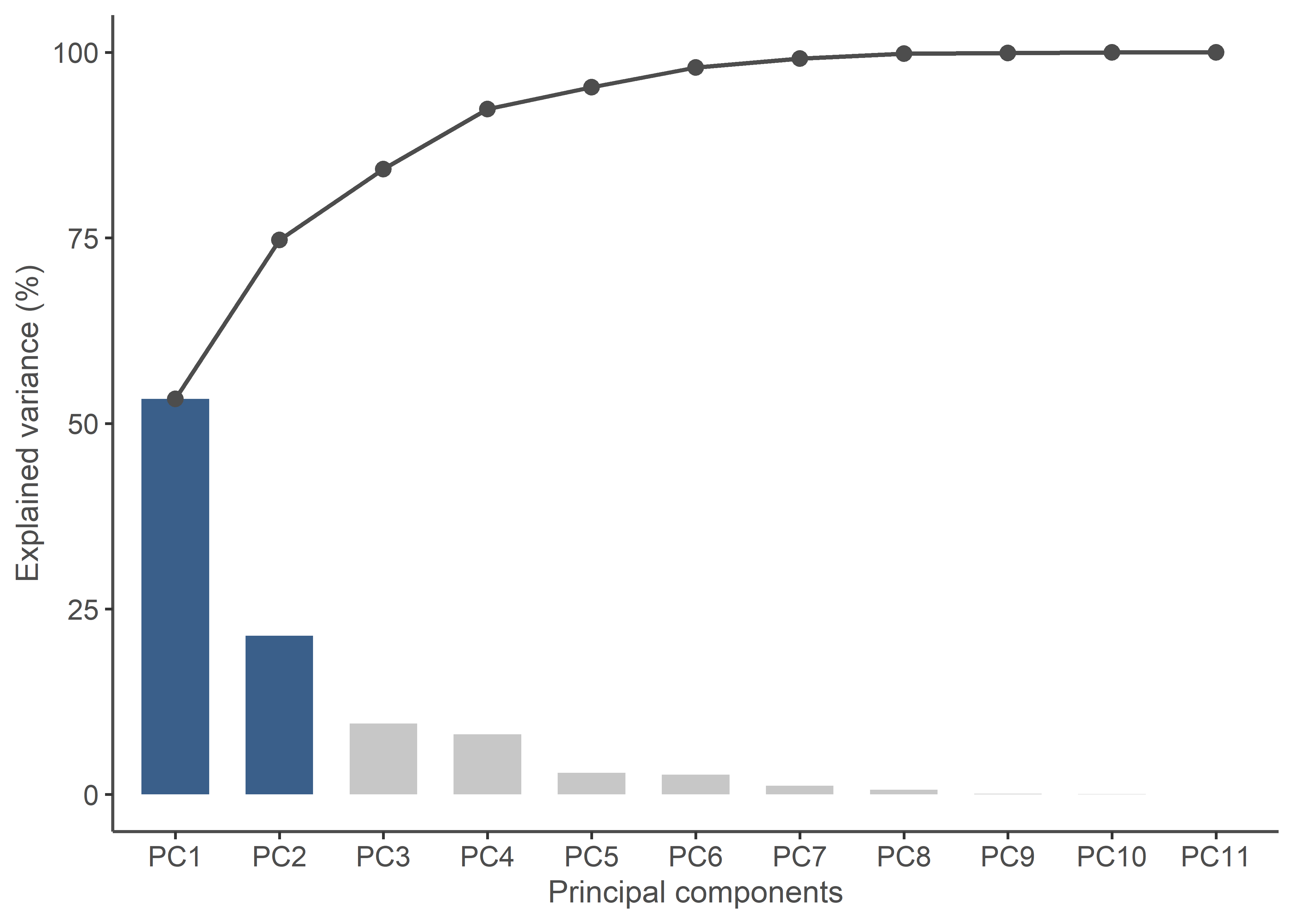
**

**Figure S2.** Scree plot showing the percentage of variance explained by each principal component in the integrative PCA of yield, compositional and mineral traits.
